# A J-Shaped Curve Relationship Between Baseline Fasting Blood Glucose and 1-Year Stroke Recurrence in Non-diabetic Patients With Acute Cerebral Infarction in Xi'an, China: A Multicenter Observational Cohort Study

**DOI:** 10.3389/fneur.2021.698793

**Published:** 2022-01-20

**Authors:** Zhongzhong Liu, Xuemei Lin, Wenjuan Lin, Qingli Lu, Pei Liu, Jing Wang, Yan Liu, Qiaoqiao Chang, Yan Wang, Chensheng Song, Fang Wang, Yaling Shi, Qing Wang, Guozheng Liu, Ye Tian, Songdi Wu

**Affiliations:** ^1^Department of Neurology, The First Affiliated Hospital of Northwest University (Xi'an No.1 Hospital), Xi'an, China; ^2^College of Life Science, Northwest University, Xi'an, China; ^3^Department of Neurology, The Affiliated Hospital of Northwest University (Xi'an No.3 Hospital), Xi'an, China; ^4^Xi'an Key Laboratory of Cardiovascular and Cerebrovascular Diseases, The Affiliated Hospital of Northwest University (Xi'an No.3 Hospital), Xi'an, China

**Keywords:** acute cerebral infarction, baseline fasting blood glucose, non-diabetic, stroke recurrence, hazard

## Abstract

**Background:**

The relationship between baseline fasting blood glucose (FBG) levels and 1-year stroke recurrence in non-diabetic patients with acute cerebral infarction (ACI) is unclear. We aimed to clarify this relationship in non-diabetic patients with ACI.

**Methods:**

Baseline FBG levels and related information of the patients were collected at admission and the events of stroke recurrence were followed up 1, 3, 6, and 12 months after the patients were discharged. Baseline FBG levels were analyzed as continuous variables and quartiles (Q1–Q4). Multivariate Cox regression models and a two-piecewise linear regression model were used to investigate the relationship and determine the threshold effect between baseline FBG levels and 1-year stroke recurrence in non-diabetic patients with ACI.

**Results:**

Overall, 1,634 non-diabetic patients with ACI were enrolled. After adjusting for potential confounding factors, the hazard is 2.24-fold higher in Q4 than those in Q2, being considered the reference in non-diabetic patients with ACI [hazard ratio (HR) = 2.24, 95%CI: 1.08–4.65, *P* = 0.031]. Plotting hazard ratios over baseline FBG levels suggested a J-shaped relationship for 1-year stroke recurrence. Further analysis revealed that the nadir value of baseline FBG levels is 4.6 mmol/L. The relationship was more significant in patients with atrial fibrillation than in those without (*P* for interaction = 0.009).

**Conclusion:**

Lower and higher baseline FBG levels may lead to an increased risk of 1-year stroke recurrence in non-diabetic patients with ACI as shown by a J-shaped curve with a nadir value of 4.6 mmol/L.

## Introduction

Acute cerebral infarction (ACI) is the most common type of stroke, accounting for higher than 70% of all strokes. It is now the second leading cause of death worldwide and the leading cause of death and disability-adjusted life-years in China (1–3).

Previous studies showed that hyperglycemia occurs in 30–40% of patients with acute ischemic stroke. Most of these patients do not have a known history of diabetes (4–6). Although some studies demonstrated that diabetes is associated with the occurrence and prognosis of stroke (7, 8), few studies have focused on blood glucose levels and prognosis of stroke in non-diabetic patients with ACI. Elevated baseline fasting blood glucose (FBG) in non-diabetic patients with acute stroke, often referred to as stress hyperglycemia, may represent the stress response of the patients and usually normalizes spontaneously after the acute phase (5). However, increasing evidence has indicated that stress hyperglycemia is not a benign phenomenon and may be associated with a heightened risk of poor prognosis (5, 9). Some studies have investigated the role of hyperglycemia in the short-term mortality prediction of patients with ACI.

In contrast, other studies have revealed the relationship between hyperglycemia and functional outcome in both diabetic and non-diabetic patients with ACI (10, 11). However, the results of previous studies may not be applicable to non-diabetic patients with stroke due to long-term high blood glucose levels. Therefore, it is clinically valuable to investigate the baseline FBG level in non-diabetic patients with ACI.

To date, few studies have examined the relationship between baseline FBG and 1-year stroke recurrence in non-diabetic patients with ACI, especially in China. Thus in the present study, we collected the baseline FBG levels and related clinical data from non-diabetic patients with ACI in Xi'an Stroke Registry Study in China, hoping to investigate the relationship between baseline FBG levels and 1-year stroke recurrence after ACI onset and to provide a more accurate reference range of baseline FBG for preventing and treating 1-year stroke recurrence in non-diabetic patients with ACI as well as a theoretical basis for individual prevention and treatment.

## Materials and Methods

### Study Population

**Figure 1:**
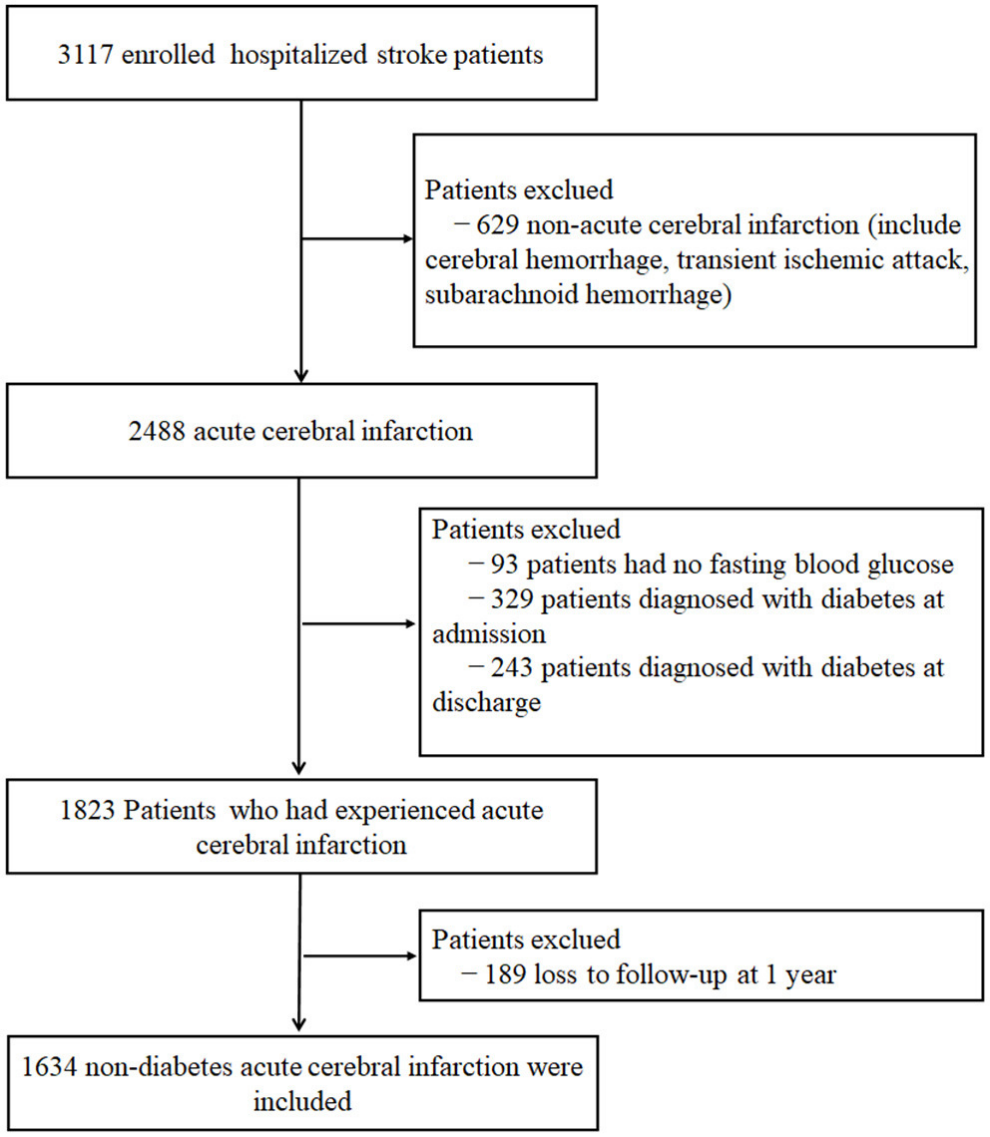
Flow chart of the screening and enrollment of study participants.

A total of 3,117 hospitalized patients with all stroke subtypes were enrolled in Xi'an Stroke Registry in China, which includes four tertiary-grade A hospitals in the Xi'an area of China from January 2015 through December. Those patients received integrated medical examinations during the initial stage of the study and were followed up at 1, 3, 6, and 12 months after symptom onset. Among these patients, 1,634 non-diabetic patients with ACI were analyzed after excluding patients with non-ACI (include cerebral hemorrhage, transient ischemic attack, and subarachnoid hemorrhage; *n* = 629), diabetes (defined as fasting blood glucose ≥7.0 mmol/L or 126 mg/dl, and receiving diabetes-related treatment) at admission (*n* = 329) or discharge (*n* = 243), missing baseline FBG value (*n* = 93) and lost during the 1-year follow-up (*n* = 189). ACI was diagnosed according to the WHO criteria and confirmed by brain MRI or CT (12). The detailed inclusion and exclusion criteria as well as the study process flowchart are shown in Figure 1. The diagnostic criteria were consistent in all participating hospitals. Patient enrollment started in January 2015, and follow-ups were completed in February 2017. The study was conducted following the guiding principles of the Helsinki Declaration. The academic committee of Xi'an No.1 hospital and the ethics committees of all participating hospitals approved the study [Approval No. 2014(5)]. All patients provided written and oral informed consent.

### Measurements and Outcomes

This multicenter prospective cohort study used data from the Xi'an Stroke Registry Study (13), a multicenter, prospective, and registry-based study to investigate the clinical characteristics and practices of stroke patients in Xi'an, China. Baseline data, including the previous medical history, assessment at admission and discharge, and laboratory test results, were collected in Table 1. The relevant factors, definitions, and criteria of previous medical history were the same as those in the Chinese Intracranial Atherosclerosis Study (14). Baseline FBG levels were measured through plasma venous blood testing within 24 h after admission (at least 8–10 h without food and only water permitted). The baseline FBG levels were analyzed and treated as continuous variables and categorical variables (quartiles Q1–Q4). FBG quartiles (Q1–Q4) were defined as the distribution of FBG from low to high and divided into four parts, on average, each part containing a quarter of the value range of FBG. The range of quartiles (Q1–Q4) was as follows: Q1: <4.53 mmol/L, Q2: 4.53–4.99 mmol/L, Q3: 5.00–5.59 mmol/L, Q4: >5.59 mmol/L. The value range of the other classification variable, body mass index, was as follows: <25 kg/m^2^, normal; 26–30 kg/m^2^, overweight; and ≥30 kg/m^2^, obese. At admission, a National Institutes of Health Stroke Scale (NIHSS) score ≤ 4 was classified as a mild deficit, 5–14 as a moderate deficit, and >14 as a severe deficit. The observed endpoint event in this study was stroke recurrence, which was defined as new acute stroke events (including cerebral infarction, cerebral hemorrhage, and subarachnoid hemorrhage) during the 1-year follow-up (15). An independent adjudication board composed of 4–5 stroke specialists from each hospital determines a new acute stroke event.

**Table 1:** Baseline and biochemical characteristics by baseline fasting blood glucose (FBG) quartiles in non-diabetes patients with acute cerebral infarction (ACI).

**Variables**	**Overall *n* = 1,634**	**Baseline FBG quartiles**				***P*-value**
		**Q1 (*n* = 405)**	**Q2 (*n* = 396)**	**Q3 (*n* = 414)**	**Q4 (*n* = 419)**	
Age (years)	64.5 ± 12.7	63.6 ± 13.7	64.5 ± 12.3	64.3 ± 12.3	64.9 ± 12.4	0.536
Male, *n* (%)	1,051 (64.3)	284 (70.1)	252 (63.6)	259 (62.6)	256 (61.1)	0.037
**Smoking**, ***n*** **(%)**						0.304
Never smoking	886 (54.2)	206 (50.9)	203 (51.3)	237 (57.2)	240 (57.3)	
Smoking cessation	328 (20.1)	92 (22.7)	81 (20.5)	76 (18.4)	79 (18.9)	
Current smoking	420 (25.7)	107 (26.4)	112 (28.3)	101 (24.4)	100 (23.9)	
Drinking, *n* (%)	426 (26.1)	110 (27.2%)	89 (22.5%)	121 (29.2%)	106 (25.3%)	0.159
**Education level**, ***n*** **(%)**						0.664
Elementary or below	802 (49.1)	214 (52.8)	197 (49.8)	197 (47.6)	194 (46.3)	
Middle school	327 (20.0)	76 (18.8)	78 (19.7)	86 (20.8)	87 (20.8)	
High school or above	505 (30.9)	115 (28.4)	121 (30.5)	131 (31.6)	138 (32.9)	
**Medical insurance type**, ***n*** **(%)**						0.185
Urban employees' medical insurance	713 (43.6)	159 (39.3)	174 (43.9)	195 (47.1)	185 (44.2)	
New type rural cooperative medical system	698 (42.7)	195 (48.1)	172 (43.4)	165 (39.9)	166 (39.6)	
Commercialinsurance	4 (0.2)	2 (0.5)	1 (0.3)	0 (0.00)	1 (0.2)	
Out-of-pocket medical	219 (13.4)	49 (12.1)	49 (12.4)	54 (13.0)	67 (16.0)	
**BMI (kg/m**^**2**^**)**, ***n*** **(%)**						0.002
Normal	1,184 (77.0)	312 (74.7)	296 (74.7)	302 (73.1)	274 (65.4)	
Overweight	391 (23.9)	85 (21.0)	91 (23.0)	96 (23.2)	119 (28.4)	
Obesity	58 (3.6)	8 (2.0)	9 (2.3)	15 (3.6)	26 (6.2)	
Prior stroke, *n* (%)	466 (28.5%)	99 (24.4)	107 (27.0)	131 (31.6)	129 (30.8)	0.048
Peripheral vascular disease, *n* (%)	46 (2.8)	15 (3.70)	12 (3.03)	12 (2.90)	7 (1.67)	0.354
Pneumonia, *n* (%)	89 (5.4)	17 (4.2)	13 (3.3)	21 (5.1)	38 (9.1)	0.001
Atrial fibrillation, *n* (%)	128 (7.8)	24 (5.9)	31 (7.8)	27 (6.5)	46 (11.0)	0.033
Hypertension, *n* (%)	1,110 (67.9)	261 (64.4)	267 (67.4)	283 (68.4)	299 (71.4)	0.203
**Stroke severity (%)**						<0.001
Mild	699 (42.8)	198 (48.9)	190 (48.0)	181 (43.7)	130 (31.0)	
Moderate	837 (51.2)	192 (47.4)	194 (49.0)	218 (52.7)	233 (55.6)	
Severe	98 (6.0)	15 (3.7)	12 (3.0)	15 (3.6)	56 (13.4)	
**Laboratory findings**						
Total cholesterol (mmol/L)	4.3 ± 1.0	4.2 ± 0.9	4.2 ± 0.9	4.4 ± 1.0	4.6 ± 1.2	<0.001
Triglycerides (mmol/L)	1.6 ± 1.3	1.5 ± 1.0	1.5 ± 0.9	1.6 ± 1.2	1.8 ± 1.9	0.002
HDL-cholesterol (mmol/L)	1.2 ± 0.3	1.2 ± 0.3	1.1 ± 0.3	1.2 ± 0.3	1.2 ± 0.3	0.410
LDL- cholesterol (mmol/L)	2.6 ± 0.8	2.4 ± 0.8	2.5 ± 0.7	2.6 ± 0.8	2.7 ± 1.0	<0.001
Fast blood glucose (mmol/L)	5.2 ± 1.1	4.2 ± 0.3	4.8 ± 0.1	5.3 ± 0.2	6.5 ± 1.2	<0.001
SBP on admission (mmHg)	145.4 ± 21.9	144.2 ± 21.7	145.6 ± 21.1	145.7 ± 22.0	145.9 ± 22.8	0.693
DBP on admission (mmHg)	85.9 ± 12.6	86.1 ± 12.9	86.3 ± 12.5	85.8 ± 12.4	85.4 ± 12.6	0.714
Alkaline phosphatase	79.2 ± 35.4	76.6 ± 25.9	77.9 ± 26.4	82.3 ± 49.9	79.9 ± 33.2	0.123
Serum creatinine (μmol/L)	74.8 ± 29.0	75.5 ± 24.3	74.5 ± 34.4	74.5 ± 31.6	74.5 ± 24.7	0.954
Blood urea nitrogen	5.1 ± 1.9	5.1 ± 2.1	5.0 ± 1.59	4.9 ± 1.98	5.2 ± 1.99	0.538
Uric acid	291.1 ± 97.1	294.6 ± 87.2	293.3 ± 97.3	288.0 ± 96.4	288.7 ± 106.3	0.718
Leukocyte count ( ×10^9^/L)	6.9 ± 2.5	6.5 ± 2.1	6.4 ± 1.8	6.9 ± 2.7	7.8 ± 2.9	<0.001

*BMI, Body Mass Index; FBG, Fasting blood glucose; NIHSS, National Institutes of Health Stroke Scale; SBP, Systolic Blood Pressure; DBP, Diastolic Blood Pressure; HLD, High-Density Lipoprotein; LDL, low-density lipoprotein*.

### Follow-Up

Patients were followed up for 1, 3, 6, and 12 months after diagnosis of ACI. The follow-up time error was no more than seven days. All enrolled patients were interviewed face-to-face or contacted over the telephone by trained research coordinators. For patients with stroke recurrence, the date of events was recorded. Patients who refused to continue participating in this study or could not be contacted by telephone after 3 attempts per day for five consecutive working days were considered as being lost to follow-up.

### Statistical Analyses

Continuous variables are presented as Mean ± SD and categorical variables are presented as percentages (%). A one-way ANOVA was used for normally distributed continuous variables to assess differences between groups; a chi-square test and trend test were used for categorical variables. When the sample did not satisfy a normal distribution, a Mann-Whitney U test was used. Multiple groups were compared using the Kruskal-Wallis rank-sum test. A Fisher's exact test was used when the theoretical frequency was <10. Confounding factors were controlled by multivariable-adjusted Cox regression models (16). The covariables in the Cox regression equation were selected according to the basis of their associations with the outcomes of interest or a change in effect estimate of higher than 10% (17). Unadjusted and multivariable-adjusted Cox regression models were used to calculate hazard ratios (HRs) and 95% confidence intervals (CIs) for the baseline FBG level and 1-year stroke recurrence relationship. The Kaplan-Meier curves (log-rank test) were used to evaluate the difference in the probability of 1-year stroke recurrence among baseline FBG quartiles. A generalized additive model was used to evaluate the non-linear relationship between baseline FBG levels and 1-year stroke recurrence in non-diabetic patients with ACI. Based on the smoothing curve, a two-piecewise linear regression model was used to identify the threshold effect. A likelihood ratio test was used to compare two different linear regression models. Subgroups analyses were conducted for pneumonia, prior stroke, atrial fibrillation and hypertension using stratified Cox regression models. The interaction among subgroups was tested using a likelihood ratio test. All analyses were performed with the statistical software packages R 3.3.2 (http://www.R-project.org, the R Foundation) and Free Statistics software versions 1.1.

## Results

### Baseline Characteristics

At the end of the one-year follow-up, 1,634 patients were not lost to follow-up while 189 were lost. By comparing the clinical characteristics of the two groups with or without patients lost to follow-up, only age, education level, and medical insurance type differed between the two groups and there was no statistical difference in other variables (Supplementary Table 1). This result shows that the main patient characteristic variables were comparable between patients lost and not lost to follow-up. The analyzed population was a good representation of the patients enrolled in our study.

A total of 1,634 eligible subjects (1,051 men, 583 women) were included in our study. The mean age was 64.5 ± 12.7 years. The mean baseline FBG level was 5.2 ± 1.1 mmol/L. The baseline demographic, clinical, and biochemical characteristics of the baseline FBG level quartile (Q1–Q4) groups were compared (Table 1). The patients with higher baseline FBG levels were more likely to be women, overweight or obese, more likely to have a prior stroke, pneumonia, or atrial fibrillation, and more likely to have a moderate or severe NIHSS score at admission. Furthermore, the baseline FBG level was directly proportional to total cholesterol, triglycerides, and LDL-cholesterol levels as well as leukocyte counts. Baseline FBG levels among quartiles showed no significant differences in age, smoking and drinking habits, education level, medical insurance type, peripheral vascular disease, hypertension, HDL-cholesterol level, systolic blood pressure at admission, diastolic blood pressure at admission, and levels of alkaline phosphatase, serum creatinine, blood urea nitrogen, or uric acid.

### Relationship Between Baseline FBG Levels and 1-Year Stroke Recurrence in Non-diabetic Patients With ACI

**Figure 2:**
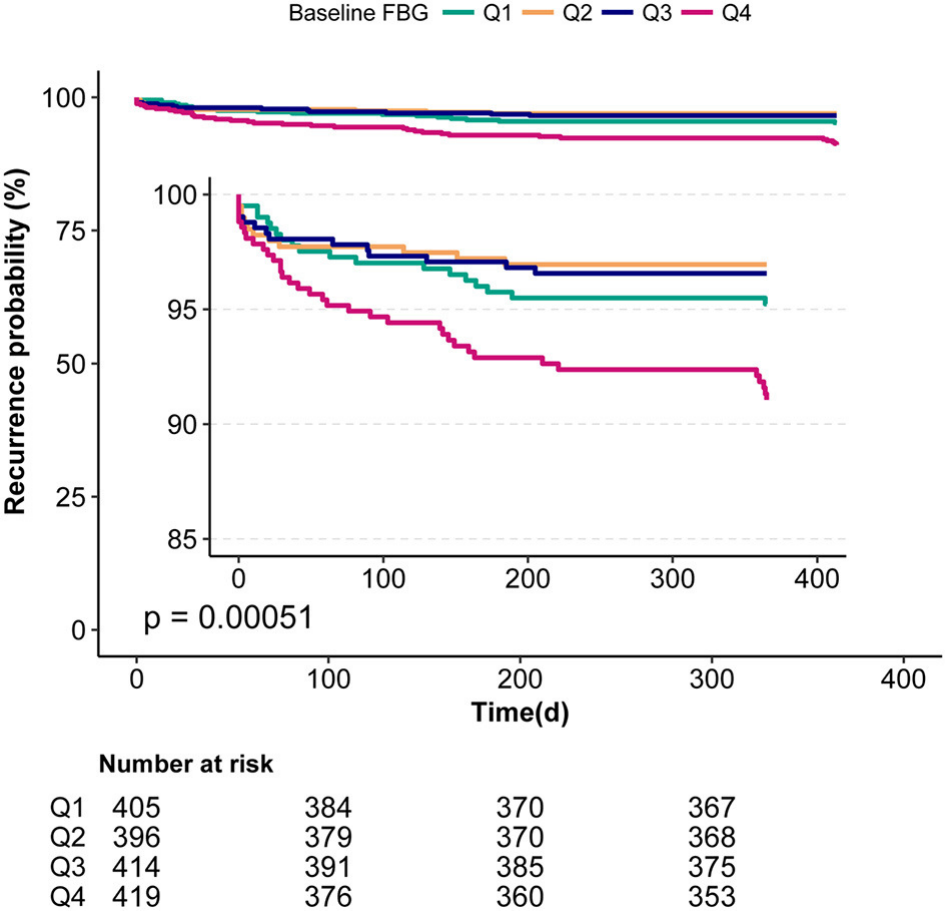
Kaplan-Meier analyses of the relationship between baseline FBG and 1-year stroke recurrence in non-diabetic patients with ACI.

Table 2 shows the results of the unadjusted and multivariable-adjusted Cox regression models. First, we analyzed with the baseline FBG level as a continuous variable. For every one mmol/L increased in the baseline FBG level, the risk of 1-year stroke recurrence in the unadjusted model and model I increased by 35 and 31%, respectively (unadjusted: HR = 1.35, 95%CI:1.19–1.53, *P* < 0.001; model I: HR = 1.31, 95%CI:1.17–1.47, *P* < 0.001). After adjusting for potential confounding factors, higher baseline FBG levels were still associated with a higher stroke recurrence in model II (HR = 1.17, 95%CI: 1.03–1.39, *P* = 0.021). Then, we analyzed the baseline FBG level as categorical variable quartiles (Q1–Q4). After adjusting for potential confounding factors, the hazard is 2.24-fold higher in Q4 than those in Q2, which was considered the reference in non-diabetic patients with ACI (HR = 2.24, 95%CI: 1.08–4.65, *P* = 0.031; Table 2). Though there was no significant difference in either Q1 or Q3 when compared with Q2, the trend test analysis showed a significant difference in the trend of increased risk in each quartile of model II (for trend *P* = 0.045). In addition, the Kaplan-Meier curve analyses also showed that the 1-year recurrence rate of stroke in Q4 was significantly higher (Figure 2).

**Table 2:** The relationship between baseline FBG and 1-year stroke recurrence in non-diabetic patients with ACI.

**Outcomes**	***n***	**Events, *n* (%)**	**Unadjusted HR (95% CI)**	***P*-value**	**Model I HR (95% CI)**	***P*-value**	**Model II HR (95% CI)**	***P*-value**
Baseline FBG (mmol/L)	1,634	81 (5)	1.35 (1.19−1.53)	<0.001	1.31 (1.17−1.47)	<0.001	1.18 (1.01−1.37)	0.036
**Baseline FBG quartiles**								
Q1 (<4.53)	405	19 (4.7)	1.55 (0.75−3.2)	0.232	1.54 (0.75−3.18)	0.239	1.76 (0.84−3.73)	0.137
Q2 (4.53–4.99)	396	12 (3)	Reference		Reference		Reference	
Q3 (5.00–5.59)	414	14 (3.4)	1.12 (0.52−2.43)	0.767	1.15 (0.53−2.49)	0.724	1.21 (0.54−2.68)	0.642
Q4 (>5.59)	419	36 (8.6)	2.95 (1.54−5.68)	0.001	2.97 (1.55−5.72)	0.001	2.28 (1.14−4.58)	0.02
*P* for trend				0.001		0.001		0.042

*Model I adjusted for age, gender. Model II adjusted for model I plus smoking, drinking, prior stroke, hypertension, pneumonia, atrial fibrillation, NIHSS score at admission, blood urea nitrogen, serum creatinine. FBG, fasting blood glucose; HR, hazard ratio; CI, confidence interval*.

### Threshold Effect Analysis of Baseline FBG Levels on 1-Year Stroke Recurrence

**Figure 3:**
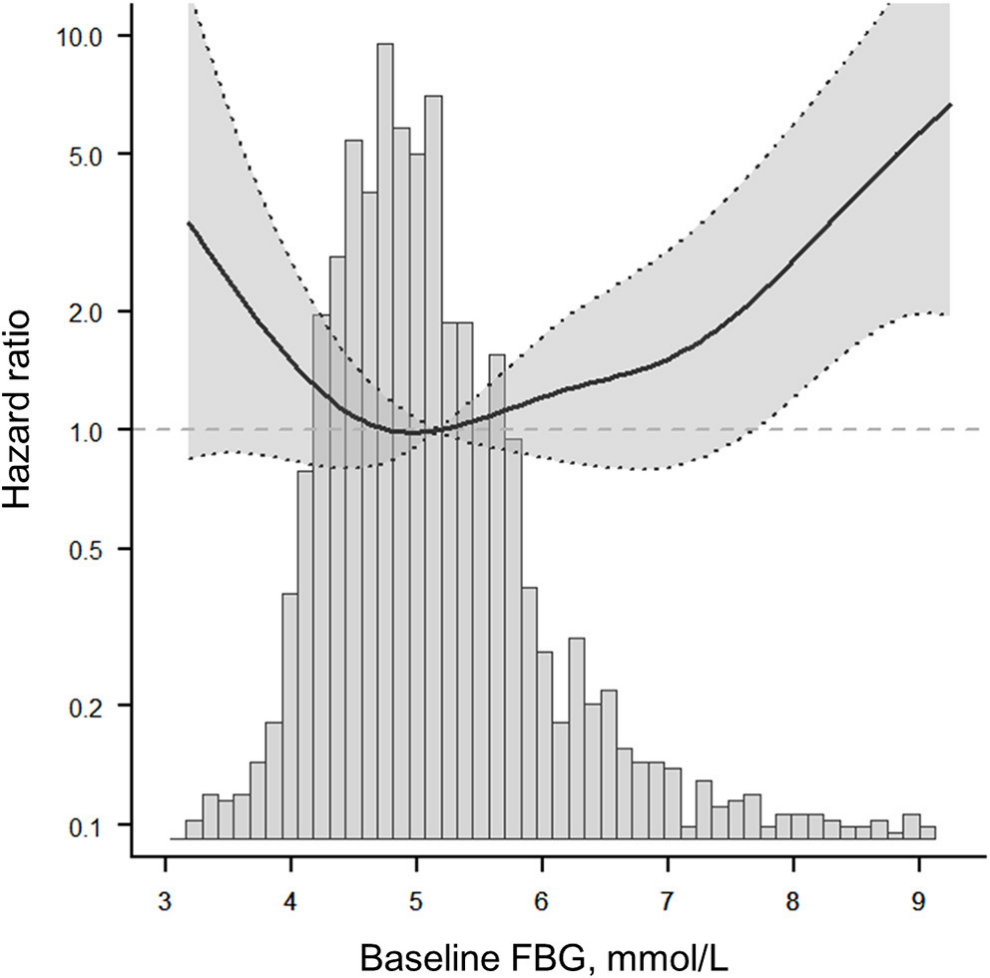
The fitted curve shows a J-shaped relationship between baseline FBG and 1-year stroke recurrence in non-diabetic patients with ACI. The solid line indicates the adjusted hazard ratio and the dashed lines the 95% confidence interval bands.

After adjusting for potential confounding factors, smoothing curve analysis showed a J-shaped curve relationship between baseline FBG levels and 1-year stroke recurrence in non-diabetic patients with ACI (Figure 3). The two-piecewise linear regression model analysis showed that the cut-off value of baseline FBG levels was 4.6 mmol/L. To the left of the cut-off value, the risk of 1-year stroke recurrence was negatively correlated with baseline FBG levels until it was 4.6 mmol/L (HR = 0.22, 95%CI:0.06–0.86, *P* = 0.029), whereas to the right of the cut-off value, the risk of 1-year stroke recurrence significantly increased (HR = 1.37, 95%CI:1.05–1.79, *P* = 0.022). The above results indicated that lower and higher baseline FBG levels were associated with high 1-year stroke recurrence (*P* = 0.004; Table 3).

**Table 3:** Threshold effect analysis of baseline FBG and 1-year stroke recurrence in non-diabetic patients with ACI.

**Outcome**	**HR (95%CI)**	***P* value**
One-line linear regression model	1.18 (1.01−1.37)	0.036
**Two-piecewise linear regression mode**		
Baseline FBG <4.6 mmol/L	0.22 (0.06−0.86)	0.029
Baseline FBG ≥4.6 mmol/L	1.37 (1.05−1.79)	0.022
Likelihood ratio test	0.004	

*Adjusted for age, gender, smoking, drinking, prior stroke, hypertension, pneumonia, atrial fibrillation, NIHSS score at admission, blood urea nitrogen, serum creatinine. FBG, fasting blood glucose; HR, hazard ratio; CI, confidence interval*.

### Subgroups Analyses

To assess whether the relationship between baseline FBG levels and 1-year stroke recurrence among different subgroups is consistent, we performed stratified analyses and interactive analyses (Table 4). The data showed that the atrial fibrillation status played an interactive role in the relationship between baseline FBG levels and 1-year stroke recurrence (for interaction *P* = 0.009). Although the differences were not significant in the trend test (*P* = 0.068), the risk of stroke recurrence in Q3 (HR = 3.82, 95%CI:0.39–37.26) and Q4 (HR = 4.24, 95%CI:0.48–37.37) were higher than that in Q2. Moreover, the baseline FBG level in Q4 was associated with 1-year stroke recurrence in patients with hypertension (HR = 2.48, 95% CI: 1.15–5.32) but not significant in interactive analyses (*P* for interaction = 0.25). Similar results were found for patients without pneumonia (HR = 2.52, 95%CI: 1.09–5.8) and without previous stroke (HR = 3.57, 95%CI: 1.17–10.9) but not significant in interactive analyses.

**Table 4:** Subgroup analyses of the association between baseline FBG quartiles and 1-year stroke recurrence in non-diabetic patients with ACI.

**Confounding factor category**	**Baseline FBG quartiles**				***P* for trend**	***P* for interaction**
	**Q1**	**Q2**	**Q3**	**Q4**		
**Hypertension**						0.25
No	4.23 (0.87, 20.5)	Reference	1.49 (0.24, 9.36)	1.39 (0.24, 8.03)	0.769	
Yes	1.3 (0.53, 3.17)	Reference	1.16 (0.48, 2.83)	2.48 (1.15, 5.32)	0.013	
**Atrial fibrillation**						0.009
No	1.88 (0.86, 4.07)	Reference	0.8 (0.32, 1.98)	1.85 (0.86, 3.98)	0.369	
Yes	0 (0, Inf)	Reference	3.82 (0.39, 37.26)	4.24 (0.48, 37.37)	0.068	
**Pneumonia**						0.529
No	2.44 (1.04, 5.72)	Reference	1.29 (0.5, 3.31)	2.52 (1.09, 5.8)	0.106	
Yes	0.69 (0.1, 4.58)	Reference	1.18 (0.25, 5.63)	1.99 (0.49, 8.14)	0.196	
**Prior stroke**						0.113
No	3.89 (1.2, 12.58)	Reference	2.8 (0.86, 9.13)	3.57 (1.17, 10.9)	0.072	
Yes	0.98 (0.32, 2.96)	Reference	0.51 (0.15, 1.78)	2 (0.79, 5.03)	0.144	

*Adjusted for age, gender, smoking, drinking, prior stroke, hypertension, pneumonia, atrial fibrillation, NIHSS score at admission, blood urea nitrogen, serum creatinine. FBG, fasting blood glucose; Q1, <4.53 mmol/L; Q2, 4.53–4.99 mmol/L; Q3, 5.00–5.59 mmol/L; Q4, >5.59 mmol/L*.

## Discussion

In this multicenter cohort study, we found that either lower or higher levels of baseline FBG were associated with an elevated risk of 1-year stroke recurrence, and the relationship between them presented as a J-shaped curve with a baseline FBG nadir value of 4.6 mmol/L in non-diabetic patients with ACI in Xi'an area.

Previous studies have reported that multiple biomarkers, such as non-HDL cholesterol (18), Vitamin D (19), irisin (20), and inflammatory factors (21), are associated with a poor prognosis of stroke. However, different biomarkers have different predictive values for poor prognosis after stroke, and their related mechanisms are also different. Elevated baseline FBG in patients with non-diabetic acute stroke, often referred to as stress hyperglycemia, may represent the stress response of patients. A previous study showed that elevated blood glucose might not appear to be directly associated with the outcome of acute ischemic stroke, and was just an epiphenomenon or marker of other underlying processes (22). However, multiple relevant studies showed that an elevated baseline FBG level appeared to be associated with infarct growth volume. Neurological deterioration increased the incidence of symptomatic intracranial hemorrhage and reduced recanalization after intravenous thrombolysis (23–27). Therefore, the relationship between baseline FBG and the outcome of stroke was still controversial.

Literature reviews found that most previous studies had pooled patients with and without diabetes together. Thus, the results could not completely rule out the effect of diabetes, even though the confounding factors were adjusted for. Although several investigations focused on the relationship between baseline FBG levels and short-term death, disability, or poor outcome of non-diabetic patients with stroke (5, 11, 28), few studies have explored the relationship between baseline FBG and stroke recurrence in non-diabetic patients with ACI in long-term follow-up (12 months). In addition, due to the variability in geographical location, lifestyle, and socioeconomic status in different countries and people, significant differences exist in the role and optimal range of the same biomarkers in different people. In this study, we explored the relationship between baseline FBG levels and 1-year stroke recurrence only in non-diabetic patients with ACI in the Xi'an area of China. The results showed that the proportion of 1-year stroke recurrence in Q4 was significantly higher (*P* < 0.05) than that in other groups. After adjusting for potential confounding factors, the hazard is 2.24-fold higher in Q4 than those in Q2, being considered as the reference in non-diabetic patients with ACI. These results imply that higher levels of baseline FBG were associated with an elevated risk of 1-year stroke recurrence in non-diabetic patients with ACI. Clinicians should be concerned with rapidly elevated baseline FBG levels since they may be associated with an even greater risk of 1-year stroke recurrence.

Furthermore, the relationship and optimal range of baseline FBG levels with respect to 1-year stroke recurrence had not been elucidated so far. To clarify this relationship, our study systematically analyzed the threshold effect and dose-response relationship between baseline FBG levels and 1-year stroke recurrence in a large-scale cohort study. The results confirmed a non-linear relationship. After adjusting for potential confounders, a J-shaped curve between baseline FBG levels and 1-year stroke recurrence was observed in non-diabetic patients with ACI (Figure 2). The optimal range of baseline FBG was in Q2 (4.53–4.99 mmol/L), and the cut-off value was 4.6 mmol/L (Table 4); values that are lower and higher than 4.6 mmol/L may lead to an increased 1-year risk of stroke recurrence in non-diabetic patients with ACI.

Our result was inconsistent with those in previous correlational studies. Ntaios found a J-shaped curve between blood glucose levels and 12-month functional outcome in patients with ischemic stroke, and initial serum glucose values between 3.7 and 7.3 mmol/L were found to be associated with a favorable outcome (29). Staszewski detected that maintaining strict glycemic control (4.5–7.0 mmol/L) is relatively safe and may improve stroke outcomes (30). Pan discovered a weak J-shaped curve between FBG levels and the risk of stroke, with an FBG nadir value of 4.9 mmol/L in minor ischemic stroke or transient ischemic attack (31). Our analysis suggests that these results may be due to differences in countries, races, regions, and study designs. Our study included non-diabetic patients with ACI in Xi'an, China, with certain regional characteristics. The findings of the above and our studies suggest that the strategies of blood glucose control in hospitalization after stroke were different in various regions and populations investigated and that clinicians should pay attention to the FBG level after hospitalization. The main strategies for non-diabetic patients with ACI are as follows: (1) baseline FBG levels in hospitalized patients should be determined as early as possible. For non-diabetic patients with ACI, an individualized blood glucose control strategy should be carried out to reduce the incidence of 1-year stroke recurrence. (2) clinicians should strengthen blood glucose control education for hospitalized patients and their families, which is helpful to strengthen the awareness of long-term blood glucose control after discharge.

Subgroup analysis revealed that the relationship between baseline FBG levels and the risk of 1-year stroke recurrence remained consistent, except in patients with hypertension and atrial fibrillation. The baseline FBG level was positively associated with 1-year stroke recurrence in the patients with hypertension (HR = 2.48, 95%CI: 1.15–5.32) in Q4 (for trend *P* = 0.013). The relationship between hypertension and the increased recurrence of stroke has been well demonstrated (32, 33). A long-term follow-up study implied that the control of hypertension is a goal for reducing stroke recurrence risk (32). Hypertension can predict the risk of stroke recurrence among young adults after ischemic stroke (33). Though there was no significance in Q4 for the patients with atrial fibrillation (HR = 4.24, 95%CI:0.48–37.37), the trend test was close to significant (for trend *P* = 0.068), possibly due to the smaller number of patients with atrial fibrillation after stratification. Previous studies showed that the relationship between atrial fibrillation and increased stroke recurrence remains controversial (34–36). In a retrospective cohort study, compared with atrial fibrillation detected before the stroke, atrial fibrillation detected after stroke is associated with a low risk of stroke recurrence (34). Ischemic strokes related to atrial fibrillation are highly prevalent, presenting with severe neurologic syndromes and associated with high risk of recurrence (35). A Greek study showed that the long-term outcome was different in stroke patients with different types of atrial fibrillation and patients with paroxysmal atrial fibrillation had lower stroke recurrence rates and mortality (36). The post-stroke comorbidities of patients were recorded, but the typing of atrial fibrillation was not performed in our study. Thus, further studies need to confirm whether atrial fibrillation has an interactive effect on the 1-year recurrence of stroke in non-diabetic patients with ACI.

The mechanisms underlying the relationship between baseline FBG levels and stroke recurrence are not fully understood. There are several probable explanations for understanding the relationship. First, an elevated baseline FBG level is often referred to as stress response in non-diabetic patients with ACI, and this results from the neuro-hormonal derangements and in?ammatory response in the acute phase (9, 37). Some patients with stroke may experience increased stroke recurrence owing to these inflammatory and neuro-hormonal derangements. Second, an elevated baseline FBG level may directly cause neurotoxicity to the ischemic penumbra, causing more neuron damage and increasing the incidence of early brain edema and risk of symptomatic intracranial hemorrhage (38–40). Third, elevated baseline FBG level occurs in non-diabetic patients with ACI who are relatively deficient in insulin. Previous studies showed that insulin resistance is independently associated with poor functional outcomes (41, 42). Finally, an oscillating baseline FBG level may cause injury to or aggravate endothelial cells and initiate a specific triggering effect for oxidative stress. These are two of the key factors leading to vascular events (43, 44). In addition, our study showed that the relationship between baseline FBG levels and 1-year stroke recurrence followed a threshold e?ect with an increased risk with baseline FBG levels >4.6 mmol/L, especially in Q4, indicating that sharp, abnormally elevated baseline FBG levels may have a more deleterious e?ect causing stroke recurrence. This finding also implicates that an abnormal baseline FBG level is a potential treatment target to improve stroke recurrence. However, this implication needs to be further verified in subsequent studies.

Our study has several limitations: (1) the four hospitals included in this study were not randomly selected, thus there was potential for selection bias; (2) all the selected centers were tertiary-grade A hospitals that may not represent the status quo of non-diabetic patients with ACI in community hospitals; (3) our stroke database did not have data on the concentration of FBG changes over time in this registration-based study; (4) glycated hemoglobin test was not included in this study, thus the patients with mild diabetes being undiagnosed previously could not be excluded. We will further improve our database in the follow-up study.

## Conclusion

Our study reveals a J-shaped curve between baseline FBG level and 1-year stroke recurrence with a nadir value of 4.6 mmol/L, indicating that both the lower and higher baseline FBG levels may lead to an increased risk of 1-year stroke recurrence in non-diabetic patients with ACI. Our findings may help physicians to assess the 1-year stroke recurrence in non-diabetic patients with ACI in the acute phase.

## Data Availability Statement

The raw data supporting the conclusions of this article will be made available by the authors, without undue reservation.

## Ethics Statement

The studies involving human participants were reviewed and approved by Ethics Committee at Xi'an No.1 Hospital. The patients/participants provided their written informed consent to participate in this study.

## Author Contributions

SW and YT had full access to all of the data in the study and takes responsibility for the integrity of the data and the accuracy of the data analysis. ZL and SW planned and designed the study. ZL contributed to the data cleaning and statistical analysis. ZL, XL, and WL wrote the manuscript. QL, PL, JW, YL, QC, YW, and CS contributed to follow-up patients and recorded the data at each stage. FW, YS, QW, and GL revised the manuscript for important intellectual content. All authors read and approved the final version of the manuscript.

## Funding

The study was supported by the Science and Technology Program of Shaanxi Province (Grant Nos. 2017SF163 and 2021SF-333), the Science and Technology Plan Major Project of Xi'an city [Grant No. 201805104YX12SF38(2)], the Science and Technology Plan Project of Xi'an city [Grant No. 20YXYJ0008(1)], and the Scientific Research Project of the Xi'an Health Commission (Grant Nos. 2020ms03, 2020yb05, and 2021yb33). The funders had no role in the design and analysis of this trial.

## Conflict of Interest

The authors declare that the research was conducted in the absence of any commercial or financial relationships that could be construed as a potential conflict of interest.

## Publisher's Note

All claims expressed in this article are solely those of the authors and do not necessarily represent those of their affiliated organizations, or those of the publisher, the editors and the reviewers. Any product that may be evaluated in this article, or claim that may be made by its manufacturer, is not guaranteed or endorsed by the publisher.
