# Supplementary material for: A J-Shaped Curve Relationship Between Baseline Fasting Blood Glucose and 1-Year Stroke Recurrence in Non-diabetic Patients With Acute Cerebral Infarction in Xi'an, China: A Multicenter Observational Cohort Study
Source: Front Neurol. 2022 Jan 20;12:698793. doi: 10.3389/fneur.2021.698793 (PMC8810505; doi:10.3389/fneur.2021.698793)
Supplement: Supplementary file 1 [file Table_1.DOCX]

Supplementary table 1: The clinical characteristics of the study group were compared with those of the lost follow-up group

| Variables | Overall  n=1823 | Not loss to follow-up  at 1 year, n=1634 | Loss to follow-up  at 1 year, n=189 | *P*-value |
| --- | --- | --- | --- | --- |
| Age (years) | 64.09±12.81 | 64.35±12.68 | 61.81±13.66 | 0.010 |
| Male, n (%) | 1176(64.51%) | 1051(64.32%) | 125(66.14%) | 0.621 |
| Smoking, n (%) |  |  |  | 0.131 |
| Never smoking | 988(54.20%) | 886(54.22%) | 102(53.97%) |  |
| smoking cessation | 376(20.63%) | 328(20.07%) | 48(25.40%) |  |
| current smoking | 459(25.18%) | 420(25.70%) | 39(20.63%) |  |
| Drinking, n (%) | 476(26.11%) | 426(26.07%) | 50(26.46%) | 0.909 |
| Education level, n (%) |  |  |  | 0.036 |
| elementary or below | 886(48.60%) | 802(49.08%) | 84(44.44%) |  |
| middle school | 380(20.84%) | 327(20.01%) | 53(28.04%) |  |
| high school or above | 557(30.55%) | 505(30.91%) | 52(27.51%) |  |
| Medical insurance type, n (%) |  |  |  | <0.001 |
| Urban employees’ medical insurance | 776(42.57%) | 713(43.64%) | 63(33.33%) |  |
| New type rural cooperative medical system | 801(43.94%) | 698(42.72%) | 103(54.50%) |  |
| [Commercial](app:ds:commercial) [insurance](app:ds:insurance) | 7(0.38%) | 4(0.24%) | 3(1.59%) |  |
| Out-of-pocket medical | 239(13.11%) | 219(13.40%) | 20(10.58%) |  |
| BMI (kg/m^2^), n (%) |  |  |  | 0.582 |
| Normal | 1324(72.67%) | 1184(72.50%) | 140(74.07%) |  |
| Overweight | 436(23.93%) | 391(23.94%) | 45(23.81%) |  |
| Obesity | 62(3.40%) | 58(3.55%) | 4(2.12%) |  |
| Prior stroke, n (%) | 526(28.85%) | 466(28.52%) | 60(31.75%) | 0.354 |
| Peripheral vascular disease, n (%) | 51(2.80%) | 46(2.82%) | 5(2.65%) | 0.893 |
| Atrial fibrillation, n (%) | 139(7.62%) | 128(7.83%) | 11(5.82%) | 0.323 |
| Hypertension, n (%) | 1233(67.64%) | 1110(67.93%) | 123(65.08%) | 0.428 |
| Pneumonia, n (%) | 96(5.27%) | 89(5.45%) | 7(3.70%) | 0.310 |
| NIHSS score on admission, n (%) |  |  |  | 0.414 |
| Mild | 778(42.68%) | 699(42.78%) | 79(41.80%) |  |
| Moderate | 931(51.07%) | 837(51.22%) | 94(49.74%) |  |
| Severe | 114(6.25%) | 98(6.00%) | 16(8.47%) |  |
| Laboratory findings |  |  |  |  |
| Total cholesterol (mmol/L) | 4.35±1.03 | 4.35±1.04 | 4.34±0.90 | 0.941 |
| Triglycerides (mmol/L) | 1.58±1.30 | 1.59±1.34 | 1.50±0.89 | 0.396 |
| HDL-cholesterol (mmol/L) | 1.15±0.32 | 1.15±0.32 | 1.14±0.31 | 0.491 |
| LDL- cholesterol (mmol/L) | 2.57±0.82 | 2.57±0.82 | 2.57±0.80 | 0.958 |
| Fast blood glucose (mmol/L) | 5.20±1.06 | 5.21±1.07 | 5.11±0.91 | 0.253 |
| SBP on admission (mmHg) | 145.14±21.62 | 145.36±21.90 | 143.26±18.98 | 0.208 |
| DBP on admission (mmHg) | 85.85±12.46 | 85.89±12.60 | 85.54±11.11 | 0.714 |
| Alkaline phosphatase (U/L) | 79.29 ± 34.69 | 79.22 ± 35.40 | 79.94 ± 27.95 | 0.789 |
| Serum [creatinine](javascript:;)（μmol/L） | 74.60±28.46 | 74.75±29.03 | 73.33±23.08 | 0.517 |
| Blood Urea Nitrogen | 5.07±1.90 | 5.07±1.92 | 5.05±1.72 | 0.889 |
| Uric Acid | 292.27±97.72 | 291.09±97.07 | 302.32±102.81 | 0.140 |
| Leukocyte count（×10^9^/L） | 6.91±2.50 | 6.93±2.51 | 6.77±2.44 | 0.413 |

Abbreviations: BMI, Body Mass Index; FBG, Fasting blood glucose; NIHSS, National Institutes of Health Stroke Scale; SBP, Systolic Blood Pressure; DBP, Diastolic Blood Pressure; HLD, High-Density Lipoprotein; LDL, low-density lipoprotein.
